# Investigating the Relationship between Ethnic Consciousness, Racial Discrimination and Self-Rated Health in New Zealand

**DOI:** 10.1371/journal.pone.0117343

**Published:** 2015-02-23

**Authors:** Ricci Harris, Donna Cormack, James Stanley, Ruruhira Rameka

**Affiliations:** 1 Te Rōpū Rangahau Hauora a Eru Pōmare, University of Otago, Wellington, PO Box 7343, Wellington, 6242, New Zealand; 2 Dean’s Department, University of Otago, Wellington, PO Box 7343, Wellington, 6242, New Zealand; Queensland University of Technology, AUSTRALIA

## Abstract

In this study, we examine race/ethnic consciousness and its associations with experiences of racial discrimination and health in New Zealand. Racism is an important determinant of health and cause of ethnic inequities. However, conceptualising the mechanisms by which racism impacts on health requires racism to be contextualised within the broader social environment. Race/ethnic consciousness (how often people think about their race or ethnicity) is understood as part of a broader assessment of the ‘racial climate’. Higher race/ethnic consciousness has been demonstrated among non-dominant racial/ethnic groups and linked to adverse health outcomes in a limited number of studies. We analysed data from the 2006/07 New Zealand Health Survey, a national population-based survey of New Zealand adults, to examine the distribution of ethnic consciousness by ethnicity, and its association with individual experiences of racial discrimination and self-rated health. Findings showed that European respondents were least likely to report thinking about their ethnicity, with people from non-European ethnic groupings all reporting relatively higher ethnic consciousness. Higher ethnic consciousness was associated with an increased likelihood of reporting experience of racial discrimination for all ethnic groupings and was also associated with fair/poor self-rated health after adjusting for age, sex and ethnicity. However, this difference in health was no longer evident after further adjustment for socioeconomic position and individual experience of racial discrimination. Our study suggests different experiences of racialised social environments by ethnicity in New Zealand and that, at an individual level, ethnic consciousness is related to experiences of racial discrimination. However, the relationship with health is less clear and needs further investigation with research to better understand the racialised social relations that create and maintain ethnic inequities in health in attempts to better address the impacts of racism on health.

## Introduction

Racism is a social system underpinned by historical and political inequalities within which racialised hierarchies systematically privileging some groups over others are produced and sustained [1], [2]. In recent years, increasing research attention has focused on racism as an important driver of health operating via direct and indirect pathways, including racially-motivated violence, as a chronic stressor with physiological and mental health impacts, and through the racialised structuring of health determinants and healthcare [3]. The body of evidence documenting relationships between racism and health has grown significantly internationally [3], [4], and represents a developing research area in New Zealand [5], [6], [7], [8], [9], [10], [11], [12]. The majority of international literature has been undertaken in the United States and has focused on racial discrimination as self-reported by individuals [3], [4]. This research has made important contributions to understanding the pathways by which racism affects health. However, self-reported racial discrimination, as measured at the level of the individual alone, is unlikely to capture the complex range of ways in which living in a racialised society may influence health.

Conceptualising the mechanisms by which racism impacts on health outcomes, drives the distribution of health risk factors, and influences access to healthcare, requires racism to be contextualised within the broader social environment, in terms of what has been referred to as the ‘racial climate’ of a society [1]. Jones [1] has posited that race consciousness, or the frequency with which people think about their race (where race is understood as a socially constructed and contingent category) is a measure of how salient race is at an individual level and of the racialised social hierarchies that exist at particular points in time and within specific contexts, as part of an assessment of the broader ‘racial climate’ [1].

It has been suggested that higher frequency of thinking about one’s race may increase the likelihood that an individual will “make decisions and choices based on their race which may or may not promote health behaviors” (p. 362, [13]). In addition, race consciousness may be capturing aspects of racial centrality or strength of identity at an individual level [14], which have been suggested to have a buffering effect on the relationship between racism and health, although the evidence for this is not consistent [15], [16]. It has also been hypothesised that among African Americans in the United States race consciousness will reflect “heightened awareness of one’s stigmatized status” (p. 2, [17]), which in turn could lead to increased vigilance or stress regarding exposure to racism [17], with flow-on health effects. Brewer et al. [17] note that less is known about what race consciousness might mean for other groups, but propose that White groups may also have psychological responses associated with race consciousness that potentially impact on health.

Although the body of research is small, there is some evidence to support the differential patterning of race consciousness by race/ethnicity in the United States, Australia and New Zealand [1], [17], [18], [19]. In the United States, for example, the distribution of race consciousness has been shown to vary between Black, Hispanic, Asian and White women [1], between African American, Hispanic and White adults in the United States [18], and between Black and White patients [17], with Black respondents reporting higher levels of race consciousness than White respondents. Data from a convenience sample in New Zealand showed that Māori (the indigenous peoples of New Zealand) were more likely to say that they thought about their race constantly than NZ European/Pākehā (the numerically-dominant ethnic group) [1], [20]. In a study with urban Indigenous peoples in Australia, more than half of participants reported that they thought about being indigenous constantly [19].

A question on race consciousness has been part of the ‘Reactions to Race’ module of the Behavioral Risk Factor Surveillance System (BRFSS) in the United States for a number of years [1], and measures of race consciousness have been included in several studies [13], [17], [18], [21], [22], [23], [24], including the 1995 *Nurses’ Health Study II* and the 1997 *Black Women’s Health Study* in the United States. These studies have tended to use a single-item question to ask about the frequency of thinking about one’s race as the measure of race consciousness, aligning with the question from the BRFSS, without reference to more specific temporal and spatial contexts (e.g. at work, at home, in one’s own community, as a child).

In anaylsis of data from the BFRSS, higher race consciousness has been found to be independently associated with perceived discrimination in health care, with those who reported thinking about their race more frequently having an increased likelihood of reporting discrimination [18], [22] and worse health status [18]. Higher race consciousness has also been associated with lower likelihood of colorectal cancer screening when comparing those who always thought about their race to those who reported never thinking about their race [13]. In contrast, race consciousness was not found to be associated with trajectories in self-rated health over time among African American adults [21].

In a recent study directly focused on examining race consciousness and its relationship to health outcomes and experiences of health care among Black and White patients in the United States [17], Black patients who ever thought about their race had higher diastolic blood pressure than black patients who never thought about their race. In contrast, White patients who ever thought about their race had a higher likelihood of reporting perceived respect from their physician and a lower likelihood of medication adherence than White patients who never thought about their race [17].

In New Zealand, there are stark ethnic health disparities for most measures of health status, health risk and protective factors, and health care access [25], [26], [27], with NZ European/Pākehā tending to enjoy the most health advantage. These disparities are longstanding and are driven by the inequitable distribution of determinants of health between ethnic groups [28]. In New Zealand, the term ‘ethnicity’ is used in preference to race in official statistics collections and in administrative datasets within the health sector. It is based on a concept of self-identified affiliation, with individuals able to identify with one or more groups that they feel they belong to. The term ethnic consciousness is therefore, more appropriate in the New Zealand context (as opposed to race consciousness), and is used in reference to the New Zealand data throughout this study.

Race/ethnic consciousness is necessarily context-specific and it is unclear how this concept is related to health and ethnic inequities in health in New Zealand. Although an ethnic consciousness question has been included in both the 2002/03 and 2006/07 New Zealand Health Surveys (NZHS), it has not been analysed to date. In addition, there has been limited examination of the relationships between ethnic consciousness and experience of racial discrimination at an individual level. The aims of the current study were, therefore, to examine the distributions of ethnic consciousness by ethnicity in New Zealand, and investigate the relationship between ethnic consciousness (how often an individual thinks about their ethnicity), self-reported experiences of racial discrimination and health. Our hypotheses were that Māori (and other non-dominant ethnic groups) would think about their ethnicity more than the dominant European ethnic group, and that higher ethnic consciousness would be associated with higher reporting of individual-level experience of racial discrimination and poorer health. We also hypothesised that ethnic consciousness may capture more subtle and systemic forms of racism and would therefore have an independent association with health over and above individual reporting of experience of racial discrimination.

## Methods

Ethics approval for the current study (secondary analysis of 2006/07 New Zealand Health Survey data) was granted by the New Zealand Health and Disability Multi-Regions Committee (MEC/10/050/EXP).

### Data source

This study undertakes secondary analysis of data from the 2006/07 adult New Zealand Health Survey (NZHS) [29]. This is a national, population-based survey of usually resident New Zealand adults (aged 15+ years) that was run at regular intervals by the Ministry of Health. It provides representative information on self-reported health status, health conditions, risk and protective factors, health service use, and population demographics. NZHS data is available to researchers as confidentialised unit record files (CURFs).

### Survey design and sampling

The 2006/07 NZHS used an area-based sampling frame with the primary sampling units being meshblocks (small areas of approximately 100 people) [29]. Meshblocks were randomly selected on a population-proportional-to-size basis. Households were randomly selected within meshblocks, and eligible individuals within households. Data was collected in face-to-face interviews between 6 October 2006 and 29 November 2007 among 12488 adults. The weighted response rate was 67.9%. Further detail on survey methodology can be found elsewhere [29].

### Key variables

**Ethnic consciousness.** Ethnic consciousness is the key predictive variable of interest in this study. The NZHS question on ethnic consciousness was adapted from the BRFSS question on race consciousness [30]. Participants were asked, “How often do you think about your ethnicity?” Response options were never, once a year, once a month, once a week, once a day, once an hour, or constantly. The distribution of ethnic consciousness responses were analysed by ethnic group. The once an hour and constantly categories were combined for this analysis as very few people responded to the once an hour category. These categories were used for descriptive analyses. For model based analyses, response categories were further reduced into ‘never/yearly’, ‘monthly/weekly’, and ‘at least daily’, similar to how race consciousness variables were grouped in the study by Crawford et al. [13].

**Self-identified ethnicity.** The NZHS uses the standard ethnicity question in line with the 2006 New Zealand Population Census. This asks people to self-identify which ethnic group or groups they feel they belong to. A number of ethnic groups are given as response options, alongside a free-text field for reporting ethnicity if it is not captured in the other options. People can choose to identify with one or more ethnic groups. For analysis, participants were grouped into five mutually exclusive ethnic groupings based on prioritisation of ethnicity (for people in multiple groups) in the following order: Māori, Pacific, Asian, Other (non-European), and European. This is one of the standard methods for outputting ethnicity in New Zealand health data [31]. The main focus of analysis in this paper is on the Māori ethnic group (all individuals who identified Māori as at least one of their ethnic groups), and European, the numerically dominant ethnic grouping in New Zealand. As European is the last group in the prioritisation, it consists of individuals who only identified with the European grouping. It is important to note that the Pacific, Asian, Other and European categories are broad aggregate ethnic categories and are made up of multiple ethnic groups. In addition, the group prioritised as ‘Other’ was very small (n = 74), and data is therefore not presented for this group as statistical inferences cannot be made.

**Racial discrimination.** Participants were asked a series of questions about their experience of racial discrimination in different settings. Self-reported ‘ever’ experience of racial discrimination in New Zealand was measured using five items: ethnically motivated physical attack, ethnically motivated verbal attack, unfair treatment on the basis of ethnicity by a health professional, in work, or when gaining housing. For descriptive data analysis and logistic regression analyses where experience of racial discrimination was the outcome variable of interest, the racial discrimination items were grouped into an overall factor of any experience of racial discrimination (yes/no) as has been done elsewhere [11]. Where racial discrimination has been included as a covariate in the health outcome model, it has been categorised into three levels (no experience, reporting experience for one item, reporting experience for two or more items).

**Health.** In order to investigate the association between ethnic consciousness and health, general self-rated health was used. Participants were asked, “In general, would you say that your health is: excellent, very good, good, fair or poor”. This measure has been widely examined and shown to be consistently associated with mortality and morbidity measures for a range of population groups [32], [33]. Such measures are also used in other New Zealand studies to examine general health for different ethnic groups (for example [34], [35], [36], [37]). Health differences were assessed by dichotomising the self-rated health variable into fair/poor health versus excellent/very good/good health.

### Other variables

Other variables adjusted for as potential confounding variables were age (15–24 yr, 25–34 yr, 35–44 yr, 45–54 yr, 55–64 yr, 65–74 yr, 75+ yr) and gender (female, male). In addition, four measures of socioeconomic position were adjusted for in multivariate models. Education was classified as no secondary education qualification, or at least a secondary education qualification. The Economic Living Standards Index short form (ELSI-SF) is based on a series of questions about a persons living standards and is categorised into: severe hardship, significant hardship, some hardship, fairly comfortable, comfortable, good, very good [38], [39]. The New Zealand Index of Socioeconomic Deprivation for Individuals (NZiDep) measures individual level deprivation and includes question on material deprivation and categorised into 5 levels (1 least deprived—5 most deprived) [40]. Area-based deprivation was measured using the New Zealand Index of Deprivation 2006 (NZDep2006) [41]. Each small area (meshblock of approximately 100 people) in New Zealand can be assigned a NZDep score that is derived from the combination (by principal component analysis) of nine variables from the 2006 Census. These variables are household income, benefit receipt, home ownership, telephone access, qualifications, car access, living in a single-parent family, employment status and living space. NZDep2006 data were analysed in quintiles (1 least deprived—5 most deprived).

### Data analysis

All data were analysed in SAS 9.2 (SAS Institute, NC) using Survey analysis based procedures. Survey design stratification and clustering are taken into account in all analyses. Survey weights were used to account for probability of selection and non-response producing estimates that are representative for the New Zealand adult population and for appropriate calculation of confidence intervals. Study participants who were missing data on outcomes or model covariates were excluded from analyses (227 missing at least one variable [1.8% of total sample]: 1 person missing self-rated health outcome; 35 more missing ethnic consciousness; 191 missing SES variables [most commonly NZDep]).

In descriptive analyses, unweighted frequencies and weighted prevalences for ethnic consciousness responses are presented to show the distributions for each ethnic group category. Other demographic variables are profiled according to the reduced three-level ethnic consciousness categories. Univariate analyses (stratified by ethnicity) examined the association between experience of racial discrimination (ever) and ethnic consciousness, and self-rated health and ethnic consciousness.

Multiple logistic regression was undertaken to examine the independent association of ethnic consciousness to any reporting of racial discrimination ever, stratified by ethnic group and adjusting for age, sex, and SES (education, ELSI, NZiDep, NZDep2006). Multiple logistic regression was also used to examine the independent association of ethnic consciousness to self-rated fair/poor health, adjusting for age, sex, ethnicity, experience of racial discrimination (in levels) and SES (education, ELSI-SF, NZiDep, NZDep2006).

In multiple regression analyses, interaction terms for ethnicity and ethnic consciousness were included. Significant interactions were present where racial discrimination was modelled as an outcome, and therefore the relationship between ethnic consciousness and experience of racism are reported stratified by ethnicity. Interaction terms between ethnic consciousness and ethnicity/experience of racism were tested and found to be not significant (using Type III tests for each set of interaction parameters) in the self-rated health model and were thus not included in the second model (*p* values > 0.162 in all models).

## Results

The distribution of reporting ethnic consciousness among the broad ethnicity groupings is shown in Table 1. In general, European respondents thought about their ethnicity the least, with 57.2% of Europeans reported that they ‘never’ think about their ethnicity compared to 27.4% of Māori, 24.0% of Asian and 20.8% of Pacific adults. Only 1.9% of European participants reported thinking about their ethnicity constantly compared to 33.6% of Pacific, 24.0% of Asian and 18.6% of Māori people. The general distribution of ethnic consciousness was skewed among Europeans towards never thinking about their ethnicity, while for Māori, Pacific and Asian the distribution was more even across categories and perhaps somewhat U-shaped.

**Table 1:** Ethnic consciousness by self-identified prioritised ethnicity.

Prioritised Ethnicity	Ethnic consciousness					
	Never	At least once a year	At least once a month	At least once a week	At least once a day	Constantly / At least once an hour
	Unweighted frequency, weighted percentage (95% CI)					
European (n = 6857)	4028	1302	715	450	202	160
	57.2% (55.7, 58.7)	20.2% (19.0, 21.3)	10.8% (9.9, 11.7)	6.7% (6.0, 7.4)	3.2% (2.7, 3.8)	1.9% (1.5, 2.2)
Māori (n = 3150)	912	436	404	386	367	645
	27.4% (25.3, 29.5)	15.4% (13.5, 17.2)	13.9% (12.0, 15.7)	13.2% (11.6, 14.8)	11.5% (10.2, 12.9)	18.6% (16.8, 20.3)
Pacific (n = 915)	183	88	124	115	93	312
	20.8% (17.2, 24.5)	8.7% (6.6, 10.8)	13.6% (10.9, 16.3)	13.3% (10.5, 16.1)	9.9% (7.9, 12.0)	33.6% (28.9, 38.3)
Asian (n = 1457)	335	185	212	177	187	361
	24.0% (21.1, 26.8)	12.7% (10.5, 15)	14.4% (12.2, 16.6)	12.0% (9.9, 14)	13.0% (10.8, 15.1)	24.0% (21.2, 26.8)

Note: Data for the ‘other’ ethnic category is not shown because of small numbers.

Table 2 shows the weighted prevalences of sociodemographic variables by ethnic consciousness categories. In addition to non-European ethnicity, higher ethnic consciousness also tended to be associated with younger age and more disadvantaged socioeconomic position in descriptive analyses.

**Table 2:** Unweighted frequency and weighted prevalence of demographic variables by ethnic consciousness.

Ethnic consciousness					
Factor	Never/yearly	Monthly/weekly	At least daily		Total (across EC)
Level	Unweighted frequency,weighted % in EC cat (95% CI)				Unweighted freq, weighted %
Total in EC category	(n = 7500)	(n = 2602)	(n = 2351)		(n = 12453)
**Age group**					
15–24	906	426	328		1660
	17.2% (15.8, 18.6)	18.1% (16.0, 20.3)	19.8% (17.2, 22.4)		17.7% (16.6, 18.9)
25–34	1100	492	486		2078
	15.3% (14.2, 16.4)	17.3% (15.3, 19.2)	20% (17.8, 22.3)		16.3% (15.4, 17.2)
35–44	1404	573	595		2572
	18.6% (17.6, 19.7)	20.9% (19.0, 22.8)	22.1% (20.0, 24.2)		19.5% (18.7, 20.4)
45–54	1219	446	413		2078
	17.5% (16.4, 18.6)	19.7% (17.6, 21.8)	16.7% (14.8, 18.6)		17.8% (16.9, 18.8)
55–64	1144	316	262		1722
	14.0% (13.1, 14.9)	13.1% (11.5, 14.7)	10.5% (9.0, 11.9)		13.4% (12.6, 14.1)
65–74	919	205	173		1297
	9.5% (8.7, 10.2)	6.3% (5.3, 7.4)	7.3% (6.0, 8.7)		8.6% (8.0, 9.1)
75+	808	144	94		1046
	7.9% (7.2, 8.5)	4.6% (3.7, 5.4)	3.5% (2.7, 4.4)		6.6% (6.1, 7.2)
**Gender**					
Female	4333	1475	1382		7190
	52.0% (50.6, 53.5)	51.5% (48.9, 54.1)	52.5% (49.8, 55.2)		52.0% (50.8, 53.1)
Male	3167	1127	969		5263
	48.0% (46.5, 49.4)	48.5% (45.9, 51.1)	47.5% (44.8, 50.2)		48.0% (46.9, 49.2)
**Education**					
No secondary educ.	2579	664	747		3990
	28.7% (27.3, 30.1)	20.5% (18.5, 22.4)	26.1% (23.8, 28.5)		26.7% (25.6, 27.8)
Secondary educ.	4904	1937	1598		8439
	71.3% (70.0, 72.7)	79.5% (77.6, 81.5)	73.9% (71.5, 76.2)		73.3% (72.2, 74.4)
**ELSI**					
Severe hardship	128	49	117		294
	1.0% (0.8, 1.3)	1.2% (0.7, 1.6)	4.2% (3.1, 5.3)		1.5% (1.2, 1.7)
Significant hardship	197	93	120		410
	1.5% (1.3, 1.8)	2.9% (2.1, 3.6)	3.7% (2.9, 4.5)		2.1% (1.8, 2.4)
Some hardship	367	166	191		724
	4.0% (3.5, 4.5)	5.0% (3.9, 6.1)	6.9% (5.5, 8.4)		4.6% (4.1, 5.0)
Fairly comfortable	670	310	291		1271
	8.2% (7.4, 9.0)	11.0% (9.4, 12.7)	12.0% (10.2, 13.8)		9.2% (8.5, 9.9)
Comfortable	1427	571	560		2558
	19% (17.9, 20.2)	21.8% (19.8, 23.8)	23.6% (21.3, 25.9)		20.2% (19.3, 21.1)
Good	3271	989	804		5064
	45.5% (44.1, 46.9)	39.4% (36.9, 41.9)	37.1% (34.3, 39.8)		43.2% (42, 44.4)
Very good	1350	389	232		1971
	20.7% (19.4, 22.1)	18.7% (16.6, 20.8)	12.5% (10.5, 14.5)		19.3% (18.2, 20.4)
**NZiDep**					
Level 1 (least deprived)	4954	1561	1113		7628
	69.7% (68.3, 71.1)	64.2% (61.8, 66.7)	51.4% (48.5, 54.4)		66.2% (65.0, 67.5)
Level 2	1200	476	511		2187
	16.7% (15.6, 17.8)	18.4% (16.4, 20.5)	22.3% (20.0, 24.7)		17.8% (16.9, 18.7)
Level 3	557	226	247		1030
	6.4% (5.7, 7.1)	7.7% (6.5, 9.0)	10.6% (8.6, 12.6)		7.2% (6.6, 7.8)
Level 4	503	238	301		1042
	4.8% (4.2, 5.5)	7.2% (6.0, 8.4)	10.6% (8.9, 12.3)		6.1% (5.5, 6.6)
Level 5 (most deprived)	276	98	175		549
	2.4% (2.0, 2.8)	2.4% (1.8, 3.0)	5.1% (4.0, 6.2)		2.7% (2.4, 3.1)
**NZDep2006 Quintile**					
1 (least deprived)	1343	450	215		2008
	22.9% (20.2, 25.7)	23.7% (20.2, 27.1)	11.5% (9.0, 14)		21.6% (19.0, 24.2)
2	1360	462	294		2116
	20.0% (17.5, 22.5)	20.8% (17.6, 24)	14.9% (11.7, 18.1)		19.5% (17.1, 21.9)
3	1628	472	391		2491
	22.1% (19.6, 24.6)	17.4% (14.8, 20)	18.2% (14.8, 21.6)		20.7% (18.3, 23.0)
4	1611	563	549		2723
	19.9% (17.6, 22.2)	20.6% (17.7, 23.6)	23.3% (19.8, 26.9)		20.5% (18.2, 22.8)
5 (most deprived)	1558	655	902		3115
	15.0% (13.0, 17.0)	17.5% (14.8, 20.2)	32.1% (27.7, 36.4)		17.7% (15.6, 19.8)

Descriptive analyses showed differences in the reporting of any experience of racial discrimination by ethnicity (Table 3, lowest among European at 13.5% and highest among Asian at 35.1%). However, for each ethnic grouping, the likelihood of reporting any experience of racial discrimination increased as ethnic consciousness increased. For all ethnic categories, people who infrequently (never/yearly) thought about their ethnicity were the most likely to report no experiences of racial discrimination. Descriptive analyses also showed ethnic differences in self-rated health (Table 4) although the relationship between self-rated health and ethnic consciousness was not as consistent across groups. Thinking about one’s ethnicity somewhat (monthly/weekly) was associated with lowest reports of fair/poor health among European, Māori and Asian, while among Pacific there was a more monotonic relationship between increasing ethnic consciousness and higher reporting of fair/poor health.

**Table 3:** Experience of racial discrimination (ever) by ethnicity and ethnic consciousness.

	Ethnic consciousness				
Prioritised Ethnicity	Never/yearly	Monthly/weekly	At least daily		Total (across EC)
	Unweighted frequency				
	Weighted prevalence of racism (95% CI)				
**Racial discrimination in any domain ever**					
European	560	258	87		905
	10.7% (9.7, 11.7)	22.0% (19.1, 24.8)	26.4% (20.6, 32.3)		13.5% (12.5, 14.5)
Māori	305	252	405		962
	20.8% (18.0, 23.5)	30.0% (26.2, 33.8)	40.7% (36.9, 44.6)		29.3% (27.2, 31.4)
Pacific	50	55	116		221
	15.5% (10.4, 20.7)	21.0% (14.6, 27.4)	27.0% (21.7, 32.4)		22.0% (18.6, 25.3)
Asian	143	163	224		530
	27.3% (22.3, 32.4)	40.6% (34.6, 46.5)	38.8% (33.9, 43.8)		35.1% (31.9, 38.2)

Note: Data for the ‘other’ ethnic category is not shown because of small numbers.

**Table 4:** Self-rated fair/poor health by ethnic consciousness, stratified by ethnic group.

		Ethnic consciousness				
	Self-rated health	Never/yearly	Monthly/weekly	At least daily		Total (across EC)
		Unweighted frequency of reporting fair/poor health weighted % by category (95% CI)				
Prioritised Ethnicity						
European	Fair/poor	589	113	43		745
		9.7% (8.8, 10.6)	8.4% (6.6, 10.1)	11.9% (7.6, 16.1)		9.6% (8.8, 10.4)
Māori	Fair/poor	190	88	167		445
		14.2% (11.8, 16.5)	10.3% (7.7, 12.9)	15.2% (12.8, 17.7)		13.4% (12.0, 14.9)
Pacific	Fair/poor	34	28	66		128
		10.1% (6.4, 13.9)	12.6% (6.5, 18.6)	17.0% (12.9, 21.1)		13.8% (11.3, 16.3)
Asian	Fair/poor	57	32	62		151
		11.7% (8.2, 15.2)	7.0% (4.4, 9.6)	11.1% (8.0, 14.1)		10.2% (8.3, 12.1)

Note: Data for the ‘other’ ethnic category is not shown because of small numbers.

Table 5 presents results of logistic regression analyses for the probability of reporting ever-experiencing racial discrimination according to ethnic consciousness, stratified by ethnicity. For all ethnic groups, increased ethnic consciousness was significantly associated with increased reporting of racial discrimination with a possible dose-response pattern for European, Māori and Pacific peoples but not Asian.

**Table 5:** Logistic regression model for experience of racial discrimination in any domain (ever) by ethnic consciousness, stratified by ethnic group.

	Ethnic consciousness		
Ethnicity	Never/yearly	Monthly/Weekly	At least daily
	OR	OR (95% CI)	OR (95% CI)
**Differences by Ethnic Consciousness (within ethnic group)**			
**Adjusted for age/sex**			
European	1 (Reference)	2.30 (1.89, 2.80)	3.03 (2.21, 4.16)
Māori	1 (Reference)	1.59 (1.24, 2.03)	2.62 (2.10, 3.28)
Pacific	1 (Reference)	1.48 (0.89, 2.46)	2.09 (1.29, 3.39)
Asian	1 (Reference)	1.82 (1.28, 2.58)	1.72 (1.22, 2.42)
**+ Adjustment for SES factors**			
European	1 (Reference)	2.31 (1.89, 2.83)	2.88 (2.10, 3.96)
Māori	1 (Reference)	1.55 (1.19, 2.00)	2.66 (2.11, 3.36)
Pacific	1 (Reference)	1.37 (0.81, 2.30)	1.98 (1.20, 3.26)
Asian	1 (Reference)	1.87 (1.29, 2.71)	1.71 (1.21, 2.42)

Note: SES factors include qualification, ELSI, NZDep2006, NZiDep, racial discrimination is any experience (as measured) ever, data for the ‘other’ ethnic category is not shown because of small numbers.

Table 6 shows the results of logistic regression analyses for the association between reporting of poor/fair health and ethnic consciousness. The unadjusted association for thinking about ethnicity at least daily suggested that this group had higher odds of poor/fair health compared to people who think about their ethnicity never or yearly (OR 1.38, 95% CI 1.16, 1.64). After adjusting for age, sex and ethnicity, this high ethnic consciousness group had a slightly weaker association with fair/poor health (OR 1.21, 95% CI 0.98, 1.49). Those who thought about their ethnicity moderately (monthly/weekly thinking about your ethnicity) had a marginally lower probability of fair/poor health compared to the never group (adjusted for age/sex/ethnicity OR 0.85, 95% CI 0.70, 1.03). After adjusting for SES variables and experience of racial discrimination, the OR for the frequently vs. never ethnic consciousness comparison moved to the null (OR = 1.00, 95% CI 0.79, 1.27).

**Table 6:** Logistic regression model for poor/fair health (sequential adjustment) by ethnicity and ethnic consciousness.

	Ethnic consciousness		
Model	Never/yearly	Monthly/weekly	At least daily
	OR (95% CI)	OR (95% CI)	OR (95% CI)
Unadjusted	1 (Reference)	0.86 (0.72, 1.04)	1.38 (1.16, 1.64)
+ age, sex, ethnicity	1 (Reference)	0.85 (0.70, 1.03)	1.21 (0.98, 1.49)
+ SES	1 (Reference)	0.87 (0.71, 1.08)	1.04 (0.83, 1.31)
+ experience of racial discrimination	1 (Reference)	0.86 (0.70, 1.06)	1.00 (0.79, 1.27)

Note: SES factors include qualification, ELSI, NZDep2006, NZiDep, experience of racial discrimination is categorized in levels (no experience, reporting experience for one item, reporting two or more items).

## Discussion

In this study, ethnic consciousness was found to be differentially distributed by broad ethnic grouping in New Zealand. As hypothesised, respondents from the European ethnic grouping were the least likely to report thinking about their ethnicity, with the other ethnic groupings all reporting relatively higher ethnic consciousness. Also in line with the study hypotheses, higher ethnic consciousness was associated with an increased likelihood of reporting ever experiencing racial discrimination for all ethnic groupings. Ethnic consciousness was associated with worse self-rated health after adjusting for age, sex and ethnicity. However, this difference was no longer evident after further adjustment for socioeconomic position and individual reported experience of racial discrimination.

The finding that reporting of ethnic consciousness is lower among Europeans in New Zealand aligns with the limited published literature available that shows reporting of race/ethnic consciousness to be lower amongst ‘dominant’ racial/ethnic groups [1], [17], [18]. The distribution of ethnic consciousness by ethnicity in New Zealand differed somewhat in the current study from that previously reported by Jones [1], with generally lower ethnic consciousness among Māori and European in this study compared to the findings reported by Jones [1]. This is most likely due to the different sampling approach (convenience sample in the earlier paper, compared with a random sample), although other differences include the different points in time that the data were collected, and the way ethnicity was categorised in analysis (i.e. NZ European in the earlier paper, compared with all European here). However, the overall finding that Māori think about their ethnicity more than NZ European/Pākehā is consistent across both analyses, suggesting that there is a different lived-experience of the racialised social environment in New Zealand for Māori, and for individuals from Pacific and Asian ethnic groups, when compared with European ethnic groups. The somewhat U-shaped distribution of ethnic consciousness among the non-European ethnic groups may benefit from further investigation to elucidate the meaning of this finding and whether it is related to situations where ethnicity is not salient or to reporting practices. This may include qualitative research and the inclusion of measures to assess the context in which people are reporting ethnic consciousness.

As hypothesised, higher ethnic consciousness was significantly associated with higher likelihood of reported experience of racial discrimination for all ethnic groupings, a finding that has also been documented in the United States [18], [22]. Hausmann et al. [18], [22] found that thinking about race more frequently (once a week or more) was associated with a higher likelihood of individuals reporting discrimination in health care settings.

It is possible that increased ethnic consciousness is associated with an increased likelihood of perceiving or reporting discrimination as racially-based, as well as increased experience of racial discrimination due to differences in exposure, potentially via a relationship between ethnic consciousness and strong ethnic identity or salience. Studies have found that higher levels of group affinity and ‘sense of belonging’ for African Americans are linked to increased perceptions of racial discrimination [16]. Experiences of racial discrimination may also increase the frequency with which individuals think about their race/ethnicity (i.e. reverse causation). As this study is cross-sectional, we cannot assess the directionality of the association between ethnic consciousness and experience of racial discrimination.

Our study found that higher ethnic consciousness was associated with poorer health. However, this association was no longer apparent after adjusting for socioeconomic position and experience of racial discrimination and we did not find an additional effect of ethnic consciousness on poor/fair self-rated health as we expected. Studies undertaken in the United States have found positive associations between race consciousness and health outcomes [13], [17], [18], although not for all health measures and not for all ethnic groups. It is possible that the strength of association between race/ethnic consciousness and health may differ depending on the health outcome being considered. Race/ethnic consciousness has been conceptualised as a stressor [17], and therefore the strength of associations with health outcomes may depend, to some extent, on the link between stress and health in any given condition. The strong associations found in ours and other studies [18], [22] between ethnic consciousness and racial discrimination support the inter-relatedness of these measures with the need to better understand how such measures combine to impact on health required [3].

Adding to the complexity of interpreting the findings on race/ethnic consciousness is the variation in the conceptualisation of race consciousness in the different studies. In some studies, race consciousness is included as a co-variate at the individual level, conceptualised as a measure of racial salience [18], [22] or of racial attitudes [24]. Some studies conceptualise it as a function of racial identity, or control for it as a confounding factor. In addition, the different ways in which the levels of race consciousness are categorised across the studies makes comparisons difficult, as well as the limited literature available about the validity and other psychometric properties of the measure.

It is not entirely clear what the ethnic consciousness variable is measuring in the New Zealand context. According to Cameron, the frequency with which individuals think about their race aligns with the concept of cognitive centrality in models of racial identity [42]. However, the single-item question as used in the current study only captures frequency of thinking about ethnicity (or race) but does not capture any affective or evaluative dimensions, or assessment of the significance of that group identity to the individual [42]. In an Australian context, increased frequency of thinking about their Indigeneity was found to be associated with other aspects of Indigenous identity among urban Indigenous Australians [19]. In line with understandings of social identities, including ethnicity, as multidimensional [42], the ethnic consciousness measure in this paper is inter-related with, but not a measure of, the strength of an individual’s ethnic identity. In addition, the measure does not distinguish between whether respondents are thinking about their ethnicity in positive or negative ways; nor is it clear whether it is capturing aspects of collective group solidarity, social relations between groups, or both. The measure also does not capture the variation in ethnic consciousness that might occur over time and by specific contexts, and how this may relate to both experiences of discrimination and health outcomes.

There are other limitations to this study some of which arise from secondary analysis of an existing dataset. As mentioned, the cross-sectional nature of the study limits our ability to draw conclusions about causality (albeit that the racial discrimination question asks about historical events). Individuals were grouped into aggregate ethnic categories for analyses. There is the potential that the distribution of ethnic consciousness, racial discrimination and health outcomes (as well as other variables) differed between specific ethnic groups included within the broad groupings of European, Pacific and Asian, and that these measures may be patterned by other factors such as nativity, migrant status and religion.

The measures of racial discrimination used in the study focus on some specific types of discriminatory experiences and are, therefore, unlikely to capture the range of racial discrimination that individuals are exposed to or can articulate as well as the known limitations of self-reporting such discrimination measures [43]. In addition, this study only considered one health measure, namely self-rated general health. It is possible that ethnic consciousness may have different relationships with different health measures. For example, if ethnic/race consciousness is conceptualised as a stressor, it may have relationships with health outcomes more directly linked to stress such as mental health outcomes. There may be differences in the way people from different ethnic groups perceive and report their health [44], however the differences in self-rated general health by ethnicity seen here are consistent with ethnic differences in health outcomes more broadly in New Zealand with Māori and Pacific peoples having generally worse health across a range of measures compared with European and Asian populations [25], [45], [46], [47]. While this study considered the role of experience of racial discrimination and socioeconomic status on the ethnic consciousness/health relationship, it did not consider other potential pathway variables at the individual-level such as stress and behavioural coping responses that have been identified in the racism and health literature [3].

The strengths of the study include the nationally-representative nature of the sample. It is the first study to report on ethnic consciousness in New Zealand and also adds to the limited literature considering ethnic consciousness and health for indigenous populations. It also extends the limited literature on race/ethnic consciousness and health, by directly examining the impact of reported experience of racial discrimination on relationships between ethnic consciousness and health.

## Conclusion

The finding of different patterning of frequency of ethnic consciousness by ethnicity in New Zealand suggests different experiences of racialised social environments by ethnicity.

While the association between ethnic consciousness and racial discrimination suggests that at an individual level, ethnic consciousness is related to experiences of racial discrimination, the relationship with health is less clear and needs further investigation. However, the study does provide further support for epidemiological research into health inequities that engages in a more critical and reflexive manner with constructs of race and ethnicity and interrogates the processes by which race and ethnicity have meaning in relation to health outcomes and inequities, rather than focusing on description or the problematisation of racial/ethnic groups [48], [49]. As such, it encourages analyses that seek to better elucidate the racialised social relations that create and maintain ethnic inequities in health in attempts to more effectively address the impacts of racism on health. This is necessary in progressing the limited but emerging range of interventions that aim to reduce racism, mitigate its health effects and improve ethnic health inequalities [50].
